# Books and Authors

**Published:** 1909-04

**Authors:** 


### BOOKS AND AUTHORS.

**Constipation and Intestinal Obstruction (Obstipation).** By **Samuel Goodwin Gant,** M.D., LL.D., Professor of Diseases of the Rectum and Anus, in the New York Post-graduate Medical School and Hospital; attending surgeon for Rectal Diseases at the New York Post-graduate and Saint Mary's Hospital, and the German Polyclinic Dispensary. With 250 original illustrations. Philadelphia and London: W. B. Saunders Company. 1909. (Price, $6.00 net).

The author of this work has constructed a treatise on somewhat new lines. It would astonish the old fashioned family doctor, to read a scientific work by a scientific man, advocating the drugless treatment of constipation, treating it without medicine. The old fashioned layman, too, would be surprised He wants "physic" as he calls it, and plenty of it. It should not be inferred from this that Gant gives no medicine at all in the treatment of constipation; but it is his choice to treat the condition by other means, such as psychotherapy, diet and physical measures—for example, bodily movements, massage, mechanical vibration, electricity, and the like. He thinks he can obtain quicker and more lasting benefit by non-medicinal and, when necessary, by surgical measures, hence seldom administers drugs as curative agents; but in the symptomatic treatment of constipation he resorts to medicines occasionally. Gant is explicit in the details of the surgical procedures he employs. He is an experienced operator with excellent surgical judgment which makes his statements trustworthy. It is refreshing to read a book by an author who, having already written a surgical treatise, now puts forth one advocating other means of treating certain diseases or disorders of the intestinal tract.

We must speak of the illustrations, two hundred and fifty in number, all of which are original. Seldom have we seen such a large number of original illustrations in one book that are so clearly explanatory of the text. We think this treatise will take rank as one of the exceptionally valuable additions to the medical literature of the year 1909. At any rate it sets a pace in that regard that will serve as a challenge to others, and one which will be difficult to out run.

---

**A Textbook of Genitourinary Diseases,** including Functional Sexual Disorders in Man by **Dr. Leopold Casper,** professor in the University of Berlin. Translated and edited by **Charles W. Bonney,** B.L., M.D., Assistant Demonstrator of Anatomy in Jefferson Medical College, Philadelphia, etc. Second edition revised and enlarged with 23 illustrations and 24 full page plates, of which 8 are in colors. Philadelphia: P. Blakiston's Son & Co. 1909. (Price, $5.00).

The crucial test of a first edition once withstood makes the anxiety of subsequent editions much less. When Bonney, two years and a half ago, introduced Caspar to the American profession he told us that in his belief the book of the distinguished German professor represented the best teaching of genitourinary diseases then available. His judgment proved good. In this new edition revision has been thorough, some sections having been rearranged, others amplified, and much new material added, including a few new illustrations. A number of American urethroscopes, cystoscopes, and other instruments are described, among which Brandsford Lewis's universal cystoscope, latest model, is given a prominent place both in illustration and text. Caspar calls it a clever invention. The Brandsford Lewis operative cystoscope with accessories is also illustrated and commended. The additions made by Bonney are inclosed in brackets and he has done his work well. The treatise is one of the foremost in genitourinary diseases and contains the recent advances in this special line of practice.

---

**Medical Inspection of Schools.** Russell Sage Foundation. By **Luther Halsey Gulick,** M.D., Director of Physical Training, New York Public Schools, and **Leonard P. Ayres,** General Superintendent of Schools of Porto Rico, 1906, 1908. New York Charities Publication Committee. (Cloth, $1.00.)

This is the second book published by the charities publication committee acting for the Russell Sage foundation, the first being the campaign against tuberculosis and was noticed in these columns last month. This volume is announced as one of the byproducts of the Backward Children Investigation, a research supported by Russell Sage. It is a most important publication and is well worth the attention of both doctors and teachers. It is the first volume of medical inspection of schools published in this country.

The book places before the reader in convenient form for easy reference information concerning the historical, medical, educational, administrative, and legal phases of the work of medical inspection of school children. The most extensive bibliography of the subject yet compiled is added—an important feature of special value to librarians, school authorities, and students of sociological subjects. Single copies, postpaid, $1.00. Charities Publication Committee, 105 East 22d Street, New York.

---

**Report of the Commission of 1906 to Investigate the Condition of the Blind in the State of New York. F. Park Lewis,** M.D., Buffalo, President. Transmitted to the legislature April 10, 1907.

This is a most interesting report as well as an instructive one. It shows what the blind can do when industrial environment is provided. It proves it to be the duty of the state to provide ways and means to make every capable blind person self-sustaining. The president of the commission, Dr. F. Park Lewis of Buffalo, is enthusiastic on this point, as well as upon the feasibility of preventing say 30 per cent. of the blindness that is occurring in the United States. To do this, of course, means scrupulous care of the eyes of the new born. It means that when the head emerges from the birth tract, the eyes of the newly arriving passenger should be carefully wiped with an aseptic cloth dipped in a mild antiseptic solution; it means, further, that as soon as the infant is born its eyes should be washed perfectly clean and a 1 per cent. solution of silver nitrate should be instilled in the eyes, one drop in each eye being quite sufficient. If this be done under the careful instruction given by Credé, who first advised this method, it will be found an all-sufficient prophylaxis against ophthalmia neonatorum.

Dr. Lewis is a strenuous advocate of this method and believes were it adopted as routine, it would serve to prevent quite 30 per cent. of blindness now due to infection from the birth tract of the mother.

The report is full of interest and all persons engaged in the care of the blind or interested in their welfare may find pleasure in reading the book. It is profusely illustrated with half-tone engravings.

---

**Proceedings of the American Medico-Psychological Association** at the sixty-third annual meeting held at Washington, D. C., May 7, 10, 1907. C. W. Pilgrim, M.D., Secretary. Published by the Association. 1907.

This being the oldest of the national special societies is also the largest in membership and its roll contains the names of many men of unusual ability. Its transactions, likewise, contain every year papers of excellence. This book presents twenty-four papers and the discussions on the same besides reports of committees on scientific, as well as other subjects. The president's address by Dr. Charles G. Hill of Baltimore, is of exceeding interest, the title being, "How can we best advance the study of Psychiatry?" It is a paper showing broad scholarship on the part of its author and was heartily enjoyed by the audience. Many of the papers merit reading by men interested in the care of psychiatric patients, and all contribute to a better understanding of their management.

---

**Saunders's Pocket Medical Formulary.** With an appendix containing many useful tables, and suggestions for treatment in emergency conditions. By **William M. Powell**, M.D., member of the Philadelphia pathological society, etc. Ninth edition, thoroughly revised, and enlarged. Philadelphia and London: W. B. Saunders Company. 1909. (Price, $1.75 net).

The popularity of this formulary cannot be questioned since it is now in its ninth edition. Its usefulness, too, cannot be doubted, especially for the junior practitioner. We are not of those who believe that prescriptions should always be formulated extemporaneously in the presence of the patient. This book contains many that have been tried and stood the test, each bearing the signature of the author. It is a convenient book of reference for the office table, or may be carried in the pocket, the binding being strong leather.

---

**The National Standard Dispensatory**—containing the National History, Chemistry, Pharmacy, Actions and Uses of Medicines, including those recognised in the Pharmacopeias of the United States, Great Britain and Germany, with many references to other foreign pharmacopeias. In accordance with the Eighth Decennial Revision of the U. S. Pharmacopeia, by authorisation of the convention. By **Hobart Amory Hare**, B.Sc., M.D., Professor of Therapeutics and Materia Medica in Jefferson Medical College, Philadelphia; **Charles Caspari, Jr.**, Ph.G., Phar. D., Professor of Theoretical and Applied Pharmacy in the Maryland College of Pharmacy, Baltimore; and **Henry H. Rusby**, M.D., Professor of Botany and Materia Medica in the College of Pharmacy of the City of New York; Members of the Committee of Revision of the U. S. P.; New (2d) edition, thoroughly revised. Imperial octavo, 2050 pages, with 478 engravings. Cloth, $6.00, net; full leather, $7.00, net. Thumb-letter index, 50 cents extra. Lea & Febiger, Publishers, Philadelphia and New York.

The value of this colossal work to those who in any way have to do with drugs cannot be estimated. It opens with the text of the pure food and drugs statutes of 1906, which places the law at hand for convenient reference by every owner of the book.

Of the high authority of the work emanating from such masters as Hare, Caspari, Rusby, Geisler, Kremers and Base, it is unnecessary to speak. In short, this great encyclopedia of the latest pharmacology, pharmacy and therapeutics, is recognised as the leading reference for every one concerned with drugs, their manufacture, dispensing and medicinal uses. The *general index*, of 120 three-columned pages, contains in one alphabet the names of drugs in English, French, German, Italian, Spanish and Latin, rendering it easy to find the article on any substance used in medicine by civilised nations. This applies to the minor as well as the major drugs of the world, an inestimable service peculiar to this book. The *therapeutic index*, of 20 three-columned pages, arranged under diseases, brings most suggestively to the mind of the physician every drug of value, and guides him to the directions for its use.

The standard dispensatory is recognised authority the world over, and is quoted as such everywhere that medicine is known.

---

**Transactions of the American Otological Society.** Forty-first annual meeting, held at Atlantic City, N. J., June 23, 24, 1908. Vol. XI., part 1. Edited by **Dr. J. F. McKernon,** Secretary, 62 W. 52d St., New York. Published by the Society. Mercury Publishing Co., Printers, New Bedford, Mass. 1908.

This society is the second oldest of the national special societies, its only senior being the American Medico-Psychological Association, whose annual volume is noticed elsewhere in this issue. This volume begins with an obituary notice of Dr. D. B. St. John Roosa, which contains in its opening sentence a striking error. It says Dr. Roosa died February 10, but does not say in what year; whereas, the fact is Dr. Roosa died March 8, 1908.

The volume contains the usual number of scientific papers, the most prominent being a symposium on otosclerosis. The meeting was held under the presidency of Dr. C. J. Kepp, of Newark, N. J. The secretary is Dr. James F. McKernon, of New York.

---

**Transactions of the Thirtieth Annual Meeting of the American Laryngological Association** held at Montreal, Canada, May 11, 12, 13, 1908. New York: Published by the association. The McConnell Printing Company, printers. 1908.

The annual volume put forth by this association is always welcome to our table. The book before us is no exception to the rule and contains some unusually interesting material. The president, Dr. Herbert S. Birkett, of Montreal, chose as the subject of his address "A brief account of the history of medicine in the Province of Quebec from 1535 to 1838." It is a most interesting bit of medical history, not easily obtained and forms a fitting prelude to the group of scientific papers that follow. Of these latter a "Symposium on Sinus Diseases" presented an interesting exposition of several forms of sinusitis. The paper of Dr. John O. Roe, of Rochester, on "Methods of opening the Maxillary Antrum," with presentation of a new instrument offered opportunity for an interesting discussion. Dr. Roe presented his position clearly and his ingenious instrument will simplify the technic of the operation he advocates. The entire book possesses unusual interest.

---

**Fourth Annual Report of the Henry Phipps Institute** for the study, treatment and prevention of tuberculosis. February 1. 1906 to February 1, 1907. Edited by **Joseph Walsh,** A.M., M.D. Published by the Henry Phipps Institute. 1908.

This remarkable institute, founded and endowed by Henry Phipps, Esq., issues each year a wonderful report of the work performed. It has an able medical staff, headed by Dr. Lawrence F. Flick, as medical director. This report, however, is edited by Dr. Joseph Walsh, whose name stands at the head of the medical staff. The clinical and sociological report of the year is prepared by the medical director and we cannot do better, in attempting to describe briefly the nature of the report. than to quote from Dr. Flick's opening paragraphs.

One of the functions of the Phipps Institute, says the report, is to gather clinical and sociological data for scientific deductions. With this end in view every patient is subjected to searching inquiry into his personal and ancestral history, and is given a careful physical examination at the time when he comes under treatment. Mode of life, occupation, associations, previous diseases, deformities, and even mental attitude are studied and recorded. The physical examination includes every important organ of the body, and a record is made of the healthy as well as the unhealthy part, in order that nothing may be overlooked and that the record may show that all parts of the body have been examined.

The records of each year are bound in chronological order and preserved for reference. The annual report of the institute is made from these records, and the scientific problems which are worked out by members of the staff and recorded in the annual report are, as a rule, based upon the data contained in these records.

The material of the report is of value to all interested in the study, prevention, and treatment of tuberculosis.

---

**Transactions of the thirty-fourth annual meeting of the Oregon State Medical Association** held at Portland, Oregon, July 1, to 3, 1908. **William House,** M.D., secretary.

This association is well organised and publishes its transactions in good form each year. Dr. William House, formerly of Buffalo, is the secretary, in which capacity he has served for some years. We may be pardoned for suggesting the propriety of recurring head lines at the top of each page, as they add to the appearance of the book. The president, Dr. R. C. Coffey, of Portland, chose for the subject of his address "Organisation and Centralisation of Medicine and Surgery in the Northwest." In the course of the address we observe that Dr. Coffey deals with medical education, especially with the state examining boards and medical journals as factors in the scheme of medical education. He indorses the purchase of the two journals in Oregon and their consolidation into one, this to be owned and managed by the association.

Honorable Tracy C. Becker, of Buffalo, addressed the association in the evening of the second day. His subject being "Is a System of State Medicolegal Experts Practicable or Possible? Judge Charles E. Wolverton, of the United States District Court, opened a discussion on Mr. Becker's address, displaying a broad culture and eminent legal ability. Others took part in the discussion, including Dr. William House, making it early a leading feature of the meeting, and adding very considerably to the value of the book.

